# American Medicinal Plants

**Published:** 1887-11

**Authors:** 


American Medicinal Plants. An Illustrated and Descriptive Guide to the American Plants used as Homœopathic Remedies. By Charles F. Millspaugh, M. D. Philadelphia and New York; Bœricke & Tafel.

The sixth and last fascicle of this beautiful work is just published. We have repeatedly called the attention of our readers to it, and strongly advocated its claims to their patronage. The part now before us only serves to increase our admiration, and stimulates us to renewed recommendation. W. M. J.

